# The Current Status of Hydrogen Storage Alloy Development for Electrochemical Applications

**DOI:** 10.3390/ma6104574

**Published:** 2013-10-17

**Authors:** Kwo-hsiung Young, Jean Nei

**Affiliations:** BASF/Battery Materials-Ovonic, 2983 Waterview Drive, Rochester Hills, MI 48309, USA; E-Mail: jean.nei@BASF.com

**Keywords:** metal hydride, hydrogen storage alloy, NiMH battery, alkaline fuel cell, electrochemical reaction

## Abstract

In this review article, the fundamentals of electrochemical reactions involving metal hydrides are explained, followed by a report of recent progress in hydrogen storage alloys for electrochemical applications. The status of various alloy systems, including AB_5_, AB_2_, A_2_B_7_-type, Ti-Ni-based, Mg-Ni-based, BCC, and Zr-Ni-based metal hydride alloys, for their most important electrochemical application, the nickel metal hydride battery, is summarized. Other electrochemical applications, such as Ni-hydrogen, fuel cell, Li-ion battery, air-metal hydride, and hybrid battery systems, also have been mentioned.

## 1. Introduction

**Figure 1:**
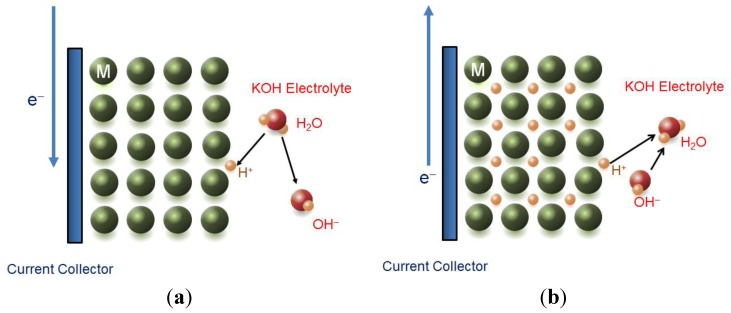
Schematics showing the electrochemical reactions between water and metal hydride during charge (**a**) and discharge (**b**). Due to the alkaline nature of the electrolyte, protons cannot desorb or absorb from the surface of metal without the incorporation of water and OH^−^.

Hydrogen storage alloys are important for a few electrochemical applications, especially in the energy storage area. The basic of electrochemical use of the hydrogen storage alloy can be described as follows: when hydrogen enters the lattice of most transition metals, interstitial metal hydride (MH) is formed. The electrons accompanying the hydrogen atoms form a metal-hydrogen band right below the Fermi level, which indicates that the interstitial MH is metallic in nature. While protons in the interstitial MH hop between neighboring occupation sites by quantum mechanical tunneling, the electrons remain within a short distance (3–10 angstroms) of the protons to maintain local charge neutrality. Under the influence of an electric field, electrons and protons will move in opposite directions. In an electrochemical environment, a voltage is applied to cause electrons to flow, and the charges are balanced out by moving conductive ions through a highly alkaline aqueous electrolyte with good ionic conductivity. During charge, a negative voltage (with respect to the counter electrode) is applied to the metal/metal hydride electrode current collector, and electrons enter the metal through the current collector to neutralize the protons from the splitting of water that occurs at the metal/electrolyte interface (Figure 1a). This electrochemical charging process is characterized by the half reaction:

M + H_2_O + e^−^→ MH + OH^−^ (1)

During discharge, protons in the MH leave the surface and recombine with OH^−^ in the alkaline electrolyte to form H_2_O, and charge neutrality pushes the electrons out of the MH through the current collector, performing electrical work in the attached circuitry (Figure 1b). The electrochemical discharge process is given by the half reaction:

MH + OH^−^ → M + H_2_O + e^−^ (2)

The standard potential of this redox half reaction depends on the chosen MH and is usually as low as possible to maximize the amount of stored energy without exceeding the hydrogen evolution potential (−0.83 V *versus* standard hydrogen electrode). Zn is an exception. With a complete 3d shell, Zn is a natural prohibitor for hydrogen evolution and thus a more negative voltage is possible, which increases the operation voltage of Ni-Zn battery.

The most important electrochemical application for MH is the negative electrode material for nickel metal hydride (NiMH) batteries. Together with a counter electrode from the Ni(OH)_2_/NiOOH system, which has been used in NiCd and NiFe batteries as early as 1901 by Thomas Edison, the NiMH battery was first demonstrated by researchers in Battelle in 1967 with a mixed TiNi + Ti_2_Ni alloy as the negative electrode [1]. Commercialization of the NiMH battery was independently realized by Ovonic Battery Company, Sanyo, and Matsushita in 1989 with AB_2_ and AB_5_ MH alloys. NiMH battery development started from small cylindrical cells (0.7 to 5 Ah) for portable electronic devices and progressed to 100 Ah prismatic cells for electric vehicle applications. The first commercially available electric vehicle in the modern era was the EV1 produced by General Motors in 1999. It was powered by a 26.4 kWh NiMH battery pack. Since then, NiMH batteries have powered more than 5 million hybrid electric vehicles made by Toyota, Honda, Ford, and other automakers, demonstrating the robustness and longevity of the NiMH battery. Recently, the NiMH battery has ventured into the stationary application market with advantages in long service life, a wide temperature range, low costs averaged over the service life, abuse immunity, and environmental friendliness. Several reviews on the topic of MH used in NiMH batteries are available [2,3,4,5,6,7,8,9,10]. In this report, we present the recent progress since the last review made in 2010 [10]. 

Besides NiMH batteries, MH (most commonly the misch metal-based AB_5_ MH alloy) can also be used in other electrochemical applications such as lithium-ion based batteries and metal-air batteries. Metal hydride electrodes have a potential window of 0.1 to 0.5 V *versus* Li+/Li and the lowest polarization among conversion electrodes. These MH electrodes have shown the capability for greater capacity and can be used as anode electrodes in lithium-ion battery [10,11,12]. An air-MH battery that utilizes a misch metal-based AB_5_ alloy in conjunction with a perovskite oxide-based cathode has been demonstrated by several research groups [13,14,15]. New types of V-flow/NiMH [16,17] and lead-acid/NiMH hybrid batteries [18] have been developed at the University of Hong Kong. Pd-treated LaNi_4.7_Al_0.3_ has been used in a Ni-hydrogen battery [19]. Another application of LaNi_5_ is the use as a cathode in a photo-electrochemical cell for water decomposition [20].

## 2. Hydrogen Storage Alloys for NiMH Battery Negative Electrodes

The use of MH in NiMH batteries started with research conducted by Schmitt and Beccu at Battelle Memorial Institute (TiNi-based) [1,21] and William and Buschow at Philips Research Laboratories (LaNi_5_-based) [2,22]. Almost 50 years of research on this subject have been conducted. In 2001, nine requirements were established for a suitable MH alloy for NiMH batteries: high capacity, good electrochemical catalysis, easy formation, excellent corrosion resistance, suitable hydrogen equilibrium, good kinetics and efficiency, long cycle life, small pressure-concentration-temperature (PCT) hysteresis, and low cost [6]. Today, the main implementation of NiMH batteries has shifted from consumer portable devices to hybrid electric vehicle and stationary applications. Accordingly, new criteria for suitable MH alloy design requirements, such as low self-discharge, good kinetics at low temperature, fast proton diffusion in the bulk, low pulverization rate during service life, and endurance of high temperature storage, are required for these applications. The amount of metallic inclusions in the MH surface oxide after activation and the high-rate dischargeability (HRD) are characteristics that are essential in meeting many of these new requirements. While the former can be quantified by magnetic susceptibility, the latter is studied by analyzing the surface reaction current density and bulk diffusion constant. Typical values from several alloy systems are compared and listed in Table 1 for reference. Recent progress of MH alloys for NiMH battery applications is reviewed in the following sub-sections categorized by alloy system.

**Table 1:** Properties comparison of several metal hydride (MH) alloys. Saturated magnetic susceptibility (*M*_S_) is proportional to the total amount of metallic nickel in the surface after activation. Applied magnetic field corresponding to half of the saturated magnetic susceptibility (*H*_1/2_) is inversely proportional to the average number of Ni atoms in a cluster. Surface exchange current (*I*_0_) and diffusion constant (*D*) are qualitative measurements of the catalytic nature of the surface reaction and the proton transportation in the bulk of the alloy, respectively.

Alloy system	Composition	*M*_S_ (memu·g^−1^)	*H*_1/2_ (kOe)	*I*_0_ (mA·g^−1^)	*D* (×10^−11^ cm^2^·s^−1^)	Reference
AB_2_	Ti_12_Zr_21.5_Ni_36.2_V_9.5_Cr_4.5_Mn_13.6_Sn_0.3_Co_2_Al_0.4_	33	0.162	32.1	9.7	[23]
AB_5_	La_10.5_Ce_4.3_Pr_0.5_Nd_1.4_Ni_60.0_Co_12.7_Mn_5.9_Al_4.7_	434	0.173	43.2	25.5	[23]
A_2_B_7_	La_16.3_Mg_7.0_Ni_65.1_Co_11.6_	369	0.125	41.0	30.8	[23]
A_2_B_7_	Nd_18.8_Mg_2.5_Ni_65.1_Al_13.6_	132	0.171	22.7	11.4	[23]
A_2_B_7_	La_3.8_Pr_7.7_Nd_7.7_Mg_4.0_Ni_72.1_Al_4.7_	314	0.128	51.5	31.9	This work
A_2_B_7_	Nd_18.4_Zr_0.2_Mg_3.6_Ni_74.1_Co_0.1_Al_3.5_	679	0.102	52.5	64	[24]
Zr-A_2_B_7_	Zr_2_Ni_7_	213	0.281	22.3	41	This work, [25]
Zr-AB_5_	ZrNi_4.5_	2286	0.400	20.1	60.6	This work, [26]

### 2.1. Rare Earth-Based AB_5_ Alloys 

**Figure 2:**
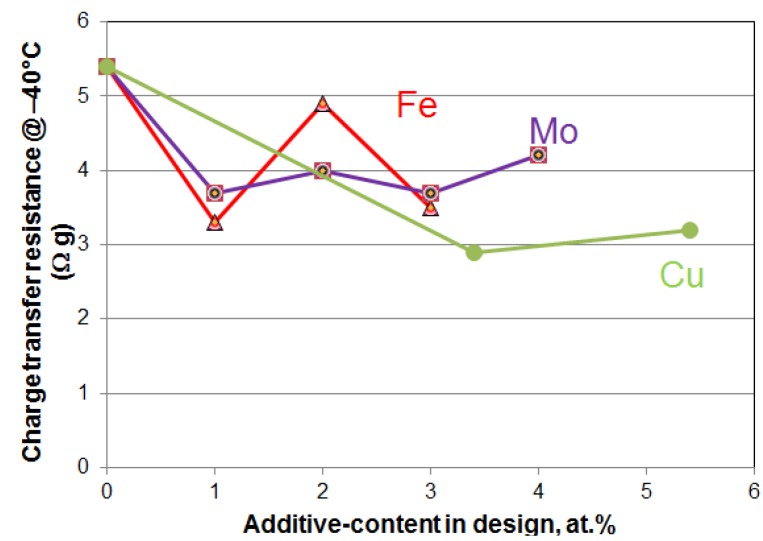
Plot of charge-transfer resistance measured at −40 °C by AC impedance as a function of Fe-, Mo-, or Cu-substitution in AB_5_ MH alloy [28,29,30]. All three additives at the lowest substitution level contribute positively in lowering the resistance.

Vucht *et al.* first reported the hydrogen storage capability of rare earth-based AB_5_ intermetallic alloy in 1970 [27]. Since then, this alloy system has become the most widely used intermetallic alloy in all metal hydride applications. After over 40 years of research, many compositions, structures, processes, and electrode fabrication modifications have been performed. Recent efforts have focused on (1) cost reduction by introducing Fe and Cu into the alloy formula to reduce/eliminate expensive Co and (2) improvement in HRD and low-temperature performance. The effects of substituting Ni by Cu, Fe, and Mo on charge-transfer resistance at −40 °C are summarized in Figure 2. The unit of Ω·g for charge-transfer resistance is used instead of the traditionally used Ω to rid the contribution from amount of electrode material; therefore, the values can be compared fairly among various MH alloys. The effect of Fe on charge-transfer resistance performance is interesting and can be explained by the evolutions of two factors: surface reaction area and catalytic ability. While surface catalytic ability is increased monotonically as the level of Fe-addition increases, surface reaction area is increased by 1% of Fe but decreased by further addition [28]. Consequently, charge-transfer resistance decreases with 1% Fe due to the improvements in both surface reaction area and catalytic ability, increases with a little higher Fe-content as the surface area diminishes, and finally decreases (but not to the level achieved with 1% Fe) with high amount of Fe when the surface catalytic ability takes on a more dominating role. Other research works on AB_5_ alloys are summarized in Table 2. 

**Table 2:** Summary of recent research on misch metal-based AB_5_ MH alloys. S: substitution, P: process, A: additives.

Method	Alloy formula/process/additives	Secondary phase (s)	Range of *x*, *etc.*	Capacity	HRD	Cycle life	Charge retention	Low temperature	Reference
S	La_10.5_Ce_4.3_Pr_0.5_Nd_1.4_Ni_64.3−*x*_ Co_5.0_ Mn_4.6_Al_6.0_Zr_0.2_Fe*_x_*	–	0 to 1.5	down	up	down	down	up	[28]
S	La_10.5_Ce_4.3_Pr_0.5_Nd_1.4_Ni_64.3_Co_8.4−*x*_Mn_4.6_Al_6.0_Cu*_x_*	(Al, Mn)Ni	0 to 5.4	down	up	down	up	up	[29]
S	La_10.5_Ce_4.3_Pr_0.5_Nd_1.4_Ni_64.3−*x*_ Co_5.0_ Mn_4.6_Al_6.0_Zr_0.2_Mo*_x_*	Mo	0 to 4	down	down	same	same	up	[30]
S	La_10.5_Ce_4.3_Pr_0.5_Nd_1.3_Ni_67.7−*x*−*y*_Mn*_x_*Al*_y_*	–	Mn (0–0.6), Al (0–3.4)	–	–	up	up	–	[31]
S	NdNi_5−*x*−*y*−*z*_Co*_x_*Al*_y_*Mn*_z_*	–	Co (0–0.5), Al (0–0.5), Mn (0–0.8)	up	down	up	down	–	[32]
S	La_0.7_Ce_0.3_Ni_3.75_Mn_0.35_ Al_0.15_Cu_0.75−*x*_Fe*_x_*	–	0 to 0.2	down	down	up	–	–	[33]
S	La_0.7_Ce_0.3_Ni_3.85_Mn_0.8_Cu_0.4_ Fe_0.15−*x*_(Fe_0.43_B_0.57_)*_x_*	La_3_Ni_12_B_2_	0 to 0.15	down	up	down	–	–	[34]
S	LaNi_3.55_Co_0.2−*x*_Mn_0.35_Al_0.15_ Cu_0.75_(Fe_0.43_B_0.57_)*_x_*	La_3_Ni_12_B_2_	0 to 0.1	down	up	down	–	–	[35]
S	LaNi_3.55_Co_0.2−*x*_Mn_0.35_Al_0.15_Cu_0.75_(V_0.81_Fe_0.19_)*_x_*	Ni-rich, La-rich	0 to 0.05	up	up	down	–	–	[36]
S	La_0.7_Ce_0.3_Ni_3.75−*x*_Mn_0.35_Al_0.15_Cu_0.75_(Fe_0.43_B_0.57_)*_x_*	–	0 to 0.15	up	up	up	–	–	[37]
S	La_0.7_Ce_0.3_Ni_3.83−*x*_Mn_0.35_Al_0.15_Cu_0.75_(Fe_0.43_B_0.57_)*_x_*	La_3_Ni_12_B_2_	0 to 0.15	down	up	down	–	–	[38]
S	La_0.7_Ce_0.3_Ni_3.75−*x*_Mn_0.35_Al_0.15_Cu_0.75_(V_0.81_Fe_0.19_)*_x_*	–	0 to 0.05	down	up	up	–	–	[39]
S	La_0.7_Ce_0.3_Ni_4.2_Mn_0.9−*x*_Cu_0.37_(V_0.81_Fe_0.19_)*_x_*	(V, Mn, Ni)	0 to 0.1	same	up	down	–	–	[40]
S	La_0.7_Ce_0.3_Ni_4.2_Mn_0.9−*x*_Cu_0.37_(Fe_0.43_B_0.57_)*_x_*	La_3_Ni_12_B_2_	0 to 0.1	down	up	down	–	–	[41]
S	La_0.7_Ce_0.3_Ni_3.75_Mn_0.35_Al_0.15_Cu_0.75−*x*_(Fe_0.43_B_0.57_)*_x_*	La_3_Ni_12_B_2_	0 to 0.1	down	up	up	–	–	[42]
S	La_0.7_Ce_0.3_Ni_3.75_Mn_0.35_Al_0.15_Cu_0.75−*x*_(V_0.81_Fe_0.19_)*_x_*	–	0 to 0.1	same	up	up	–	–	[43]
S	La_0.7_Ce_0.3_(Ni_3.65_Mn_0.35_Al_0.15_Cu_0.75_(Fe_0.43_B_0.57_)_0.10_)*_x_*	La_3_Ni_12_B_2_Ce_2_Ni_7_	0.9 to 1.0	up	up	up	–	–	[44]
S	MlNi_3.55_Co_0.75–*x*_Mn_0.4_Al_0.3_(Cu_0.75_P_0.25_)*_x_*	P-rich, Mn-rich	0 to 0.5	down	up	up then down	–	–	[45,46]
S	LaNi_5−*x*_In*_x_*	–	0.1 to 0.5	down	–	up	–	–	[47]
S	LaNi_4.3_(Co,Al)_0.7−*x*_In*_x_*	–	0 to 0.1	up	up	–	–	–	[48]
S	LaNi_4.1−*x*_Co_0.6_Mn_0.3_Al*_x_*	–	0 to 0.45	down	down	up	up	–	[49]
S	La_0.78_Ce_0.22_Ni_3.73_Mn_0.30_Al_0.17_Fe*_x_*Co_0.8−*x*_	–	0 to 0.8	down	down	up	up	–	[50]
S	LaNi_4.4−*x*_Co_0.3_Mn_0.3_Al*_x_*	–	0 to 0.2	up	Up then down	up	up	–	[51]
S	MmNi_3.70−*x*_Mn_0.35_Co_0.60_Al_0.25_B*_x_*	CeCo_4_B	0 to 0.2	down	up	–	–	–	[52]
S	La_0.35_Ce_0.65_Ni_3.54_Mn_0.35_Co_0.80−*x*_Al_0.32_Mo*_x_*	–	0 to 0.25	up	up	up	–	–	[53]
S	Mm_0.8−*x*_Ti*_x_*La_0.2_Ni_3.7_Mn_0.5_Co_0.3_Al_0.38_Mo_0.02_	–	0 to 0.05	up	–	up	up	–	[54]
S	La_0.65−*x*_Ce_0.25−*x*_Pr_0.03_Nd_0.07_Y_2*x*_Ni_3.65_Mn_0.3_Co_0.75_Al_0.3_	–	0 to 0.04	down	down	up	–	–	[55]
S	La_1−*x*_Y*_x_*Ni_3.55_Mn_0.4_Co_0.75_Al_0.3_	–	0 to 0.1	up	–	up	–	–	[56]
S	Eliminates Co, Mn	–	–	up	–	–	up	–	[57]
P	Pre-treatment if 12 M NaOH + 0.05 M NaBH_4_	–	–	–	up	up	–	up	[58]
P	Melt-spin	LaNi_3_, La_2_Ni_3_	–	up	–	down	–	–	[59]
P	Gas Atomization	–	–	down	same	up	up	same	[29]
P	Annealing temperature increase	–	–	up	down	up	up	–	[60]
A	Ni-PTFE plating	–	–	same	–	potentially up	–	–	[61]
A	Carbon nanosphere	–	–	up	up	down	–	–	[62]
A	Graphite	–	–	down	up		–	–	[63]
A	Co nano and Y_2_O_3_	–	–			up	–	–	[64]
A	Co_3_O_4_	–	–	up	up		–	–	[65]
A	Co_3_O_4_	–	–	up	up	up	–	–	[66]
A	Ni(OH)_2_	–	–	up	up	down	–	–	[67]

### 2.2. Laves Phase-Based AB_2_ Alloys 

AB_2_ alloys for NiMH battery applications are composed of main phases belonging to a family of materials known as Laves phases: hexagonal C14 phase and face-center-cubic C15 phase. C36 phase may also be present but is difficult to distinguish from C14 in XRD analysis. The main controlling factor for the C14/C15 ratio is the average electron density (*e*/*a*). Nei *et al.* reported that chemical potential can be used to fine-tune the C14/C15 threshold when Ti, Zr, and Hf, which have the same number of outer-shell electrons, are used together [68]. The common minor phases are Zr_7_Ni_10_, Zr_9_Ni_11_, ZrNi, and TiNi. The microstructures [69,70,71,72] and the contributions [73,74,75,76,77] of these phases were studied extensively in recent years.

**Figure 3:**
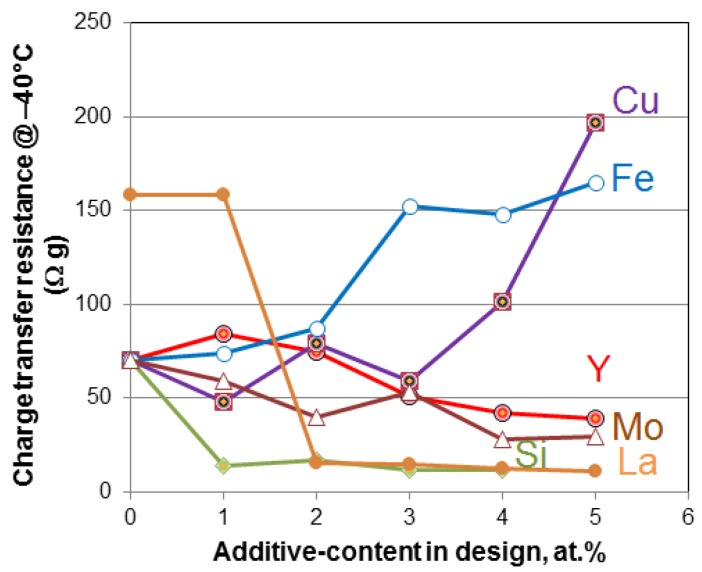
Plot of charge-transfer resistance measured at −40 °C by AC impedance as function of Cu-, Fe-, Y-, Mo-, La-, or Si-substitution in AB_2_ MH alloy [91,92,93,94,97,102]. La- and Si-modified alloys demonstrate the lowest resistance.

While many studies on AB_2_ alloys were reported, most of them focused on the influences of A- or B-site substitutions on electrochemical properties. These results are summarized in Table 3. The effect of partial substitution of Ni by various modifiers on −40 °C charge transfer resistance is summarized in Figure 3.

As shown in Figure 3, both La and Si are very effective in improving the low-temperature performance. Additionally, the influence of stoichiometry (B/A ratio) on electrochemical properties was reported [78,79]. PCT hysteresis was correlated to the pulverization rate in AB_2_ alloys [80,81,82]. Gas atomization and hydrogen annealing were introduced to produce AB_2_ metal powder directly [83,84]. V-free AB_2_ alloys were also developed to reduce raw material costs [85,86]. 

**Table 3:** Summary of recent research on Laves phase-based AB_2_ MH alloys.

Base alloy	Substitution	Major effects	Reference
C14-domintaed	Al	Al improves bulk diffusion and surface reactivity. Al and Co together improves all electrochemical performances	[87,88]
C14-domintaed	B	B improves HRD and low-temperature performance but decreases charge retention, capacity, and cycle life	[85]
C14-domintaed	C	C increases HRD and charge retention but decreases low-temperature, capacity and cycle life	[85]
C14-domintaed	Co	Co provides easy activation, improves/decreases capacity, better cycle life and charge retention, but impedes HRD	[87,89,90]
C14-domintaed	Cr	Cr improves charge retention but impedes HRD	[89]
C14-domintaed	Mo	Mo improves HRD, low-temperature performance, charge retention, and cycle life	[91]
C14-domintaed	Cu	Cu increases capacity, facilitates activation, but decreases HRD.	[92]
C14-domintaed	Fe	Fe facilitates activation, increases total electrochemical capacity and effective surface reaction area, decreases HRD and bulk diffusion, and deteriorates low-temperature performance	[87,93]
C14-domintaed	Gd	Gd improves low-temperature performance, but decreases charge retention, HRD, capacity, and cycle life	[85]
C14-domintaed	La	La improves capacity, HRD, and low-temperature performance with a trade-off of inferior cycle stability	[94]
C14-domintaed	Mg	Mg improves charge retention, deteriorates capacity, low-temperature performance, and cycle life	[85]
C14-domintaed	Mn	Mn increases capacity, facilitates activation, but decreases cycle life	[89,95]
C14-domintaed	Ni	Ni improves cycle life and HRD but reduces capacity	[89]
C14-domintaed	Pt	Pt improves capacity and HRD	[96]
C14-domintaed	Si	1 at % of Si is beneficial to HRD and low-temperature performance	[97]
C14-domintaed	Sn	Sn improves charge retention but deteriorates HRD and cycle life	[87,98]
C14-domintaed	Ti	Ti increases HRD and facilitates activation	[99]
C14-domintaed	V	V increases capacity but decreases HRD and charge retention	[100]
Both C14- and C15-dominated	Y	Y improves activation, HRD, and low-temperature performance by increasing reaction surface area	[101,102]
C14-domintaed	Zr	Zr increases capacity	[99]

### 2.3. Superlattice A_2_B_7_-Type (A_2_B_4_-AB_5_-Hybrid-Type, such as AB_3_, A_2_B_7_, A_5_B_19_, and AB_4_) Alloys 

**Figure 4:**
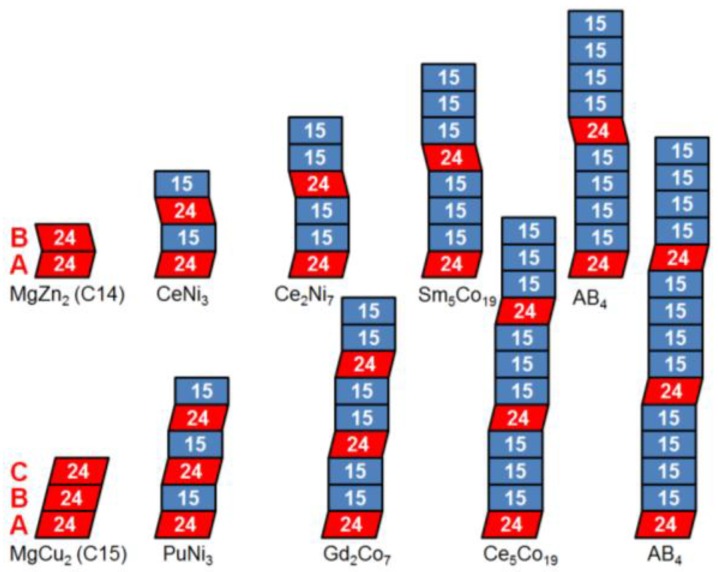
Schematics of stacking sequences of superlattice alloy systems. The stacking sequence is constructed with one to four AB_5_ (blue 15) slabs in between slabs of A_2_B_4_ (red 24). Two structures are available for each stacking sequence depending on the direction of the A_2_B_4_ slab shifts. The tilted stacking of A_2_B_4_ in a C14 structure first shifts (1/3, 1/3) and then shifts back (−1/3, −1/3) while C15 structure shifts (1/3, 1/3) consecutively on the *a*-*b* plane.

Mg-containing superlattice alloys have been used extensively in Japanese-made NiMH batteries for retail market since the introduction by Sanyo (eneloop) [103,104,105]. Batteries with superlattice A_2_B_7_-type alloys as negative electrode exhibit much lower self-discharge compared to ones with traditionally used AB_5_ alloys [105]. Therefore, NiMH battery’s storing and ready-to-use-out-of-the-pack capabilities can be much improved by the use of superlattice alloy, which enable NiMH battery to grow much more competitive compared to primary battery. Furthermore, superlattice alloys have been reported to have superior capacity, cycle life, and high-rate performances compared to the conventional alloys and are becoming a dominating force in both consumer and hybrid electric vehicle markets. The multi-phase MH system, with an overall B/A ratio between 3 and 4, is composed of a number of AB_5_ slabs placed between two A_2_B_4_ slabs (Figure 4). Mg is needed to lower the average metal-hydrogen bond strength in order to obtain the appropriate heat of hydride formation suitable for NiMH battery applications (≈ −39 kJ·mol^−1^ H_2_ at room temperature and 1 atm), and its replacement amount for rare earth is about 30% and 15% for La and Nd, respectively. Mg mainly replaces rare earth elements on the A_2_B_4_ slab. The distribution of Mg (which has a high vapor pressure during melting) in the alloy is particularly important because Mg-lean regions have a tendency to form AB_5_ phase. Lin *et al.* presented a brief review of different types of A_2_B_7_ alloys [106]. The superlattice MH alloys can be classified into three groups: La-only, La-Pr-Nd, and Nd-only. The addition of Ce promotes AB_5_ phase and therefore is rarely used. The La-only group has the highest capacity but the shortest cycle life due to the easy oxidation of La. The Nd-only group has the best charge retention and cycle life performance but the lowest capacity. Properties of the La-Pr-Nd group fall between the properties for La- and Nd-only alloys. Annealing is the most convenient method to manipulate phase distribution, and its effect was reported on La-only [107,108,109,110,111,112,113], Nd-only [24], La-Gd [114], La-Nd [115], La-Pr-Nd [116,117], and La-Ce-Pr-Nd [118] alloys. While Nd-only and La-Pr-Nd superlattice alloys are used in low self-discharge and high capacity NiMH batteries, respectively, La-only superlattice alloy is not used in commercial products due to its low cycle life from pulverization [119]. However, La-only alloy is studied extensively in the research society [120] due to easy sample preparation. The recent progress in the NiMH battery applications of superlattice alloys is summarized in Table 4.

**Table 4:** Summary of recent progress in electrochemical property improvement in superlattice MH alloys.

Substitution/Process	Alloy formula	Range of *x*	Capacity	HRD	Cycle life	Charge retention	Comment	Reference
Ce	(La_0.7_Mg_0.3_)_1−*x*_Ce*_x_*Ni_2.8_Co_0.5_	0 to 0.1	down	up	up	–	–	[121]
Dy	(La_1−*x*_Dy*_x_*)_0.8_Mg_0.2_Ni_3.4_Al_0.1_	0 to 0.2	up	–	same	down	–	[122]
Gd	(La_2−*x*_Gd*_x_*Mg)(NiCoAlZn)_3.5_	0 to 1	up	down	up	–	–	[123]
Nd	La_0.8−*x*_Nd*_x_*Mg_0.2_Ni_3.35_Al_0.1_Si_0.05_	0 to 0.2	up	up	up	–	–	[124]
Nd	(La_1−*x*_Nd*_x_*)_2_Mg(Ni_0.8_Co_0.15_Mn_0.05_)_9_	0 to 0.3	down	–	up	–	–	[125]
Pr	La_0.75−*x*_Pr*_x_*Mg_0.25_Ni_3.2_Co_0.2_Al_0.1_	0 to 0.4	–	–	up	–	–	[126]
Pr	La_0.8−*x*_Pr*_x_*Mg_0.2_Ni_3.15_Co_0.2_Al_0.1_Si_0.05_	0 to 0.3	up	up	up	–	–	[127]
Pr	La_0.75−*x*_Pr*_x_*Mg_0.25_Ni_3.2_Co_0.2_Al_0.1_	0 to 0.2	down	–	up	–	–	[128]
Sc	(La_2−*x*_Gd*_x_*Mg)(NiCoAlZn)_3.5_	0 to 1	up	up	same	–	–	[123]
Sm	La_0.8−*x*_Sm*_x_*Mg_0.2_Ni_3.15_Co_0.2_Al_0.1_Si_0.05_	0 to 0.1	up	up	up	–	–	[129]
Ti	(La_0.67_Mg_0.33_)_1−*x*_Ti*_x_*Ni_2.75_Co_0.25_	0 to 0.05	down	up	up	–	–	[130]
Ti	(La_1−*x*_Ti*_x_*)_2_MgNi_8.25_Co_0.75_	0 to 0.1	down	up	up	–	–	[131]
Zr	La_0.75−*x*_Pr*_x_*Mg_0.25_Ni_3.2_Co_0.2_Al_0.1_	0 to 0.2	up	–	up	–	–	[128]
Zr	La_0.75−*x*_Zr*_x_*Mg_0.25_Ni_3.2_Co_0.2_Al_0.1_	0 to 0.2	–	down	–	–	–	[132]
Mg	La_1.7+*x*_Mg_1.3−*x*_(NiCoMn)_9.3_	0 to 0.4	up	down	down	–	Improves activation	[133]
Mg	La_0.85_Pr_0.15_Mg*_x_*(Ni_0.7_Co_0.2_Mn_0.1_)_9_	0.5 to 1.0	up	up	–	–	–	[134]
Mg	La_0.8−*x*_Gd_0.2_Mg*_x_*Ni_3.1_Co_0.3_Al_0.1_	0.1 to 0.15	up	–	up	–	–	[135]
Mg	La_0.8−*x*_Gd_0.2_Mg*_x_*Ni_3.1_Co_0.3_Al_0.1_	0 to 0.15	up	–	up	–	–	[136]
Mg	La_0.8−*x*_Gd_0.2_Mg*_x_*Ni_3.3_Co_0.3_Al_0.1_	0 to 0.15	up	up	up	–	–	[137]
Ca	La_0.67_Mg_0.33−*x*_Ca*_x_*Ni_2.75_Co_0.25_	0 to 0.05	–	up	up	–	–	[138]
Al	La_0.75_Mg_0.25_Ni_3.5−*x*_Co_0.2_Al*_x_*	0 to 0.09	down	down	up	–	–	[139]
Co	LaNi_3.2−*x*_Mn_0.3_Co*_x_*	0.2 to 0.8	down	–	up	up	–	[140]
Co	La_0.7_Zr_0.1_Mg_0.2_Ni_3.4−*x*_Co*_x_*Fe_0.1_	0.15 to 0.25	down	up	up	–	–	[141]
Co	La_0.55_Pr_0.05_Nd_0.15_Mg_0.25_Ni_3.5−*x*_Co*_x_*Al_0.25_	0 to 0.3	up	up	same	–	–	[142]
Co + Al	La_0.45_Pr_0.135_Nd_0.315_Mg_0.1_Ni_3.9_Al_0.2_	0 to 0.1	down	up	up	–	–	[143]
Co + Al	La_2_MgMn_0.3_Ni_8.7−*x*_(Co_0.5_Al_0.5_)*_x_*	0 to 2	down	up	up	–	–	[144]
Co + Al	La_0.55_Pr_0.05_Nd_0.15_Mg_0.25_Ni_3.5_(Co_0.5_Al_0.5_)*_x_*	0 to 0.3	up	–	up	up	–	[145]
Al	LaNi_3. 8–*x*_Al*_x_*	0 to 0.4	up then down	–	–	–	Improves activation	[146]
Mn	(La_0.8_Nd_0.2_)_2_Mg(Ni_0.9−*x*_ Co_0.1_Mn*_x_*)_9_	0 to 0.1	up	–	up	–	–	[147]
Mn	La_0.78_Mg_0.22_(Ni_0.9−*x*_ Co_0.1_Mn*_x_*)_3_	0 to 0.01	down	up	down	–	–	[148]
Cu	LaMg_2_Ni_9–*x*_Cu*_x_*	0 to 9	down	–	–	–	–	[149]
Si	La_0.8_Mg_0.2_Ni_3.3_Co_0.52_Si*_x_*	0 to 0.1	down	up	up	–	–	[107]
Ni	CeMn_0.25_Al_0.25_Ni_1.5+*x*_	0 to 1.1	up	–	–	–	–	[150]
H_2_O_2_ in electrolyte	Nd_18.8_Mg_2.5_Ni_75.1_Al_3.6_	–	up		up	up	–	[151]
Melt-spin	La_0.75−*x*_Zr*_x_*Mg_0.25_Ni_3.2_Co_0.2_Al_0.1_	0 to 0.2	–	up	–	–	–	[132]
Melt-spin	La_2_MgNi_9_	–	–	–	–	–	Improves Mg-homogeneity	[152]
Ball milling	La_0.7_Mg_0.3_Ni_2.8_Co_0.5−*x*_Fe*_x_*	0 to 0.5	up	–	up	–	–	[153]
NiCuP plating	La_0.88_Mg_0.12_Ni_2.95_Mn_0.10_Co_0.55_Al_0.10_	–	up	up	–	up	–	[154]
Spark plasma sintering	La_0.85_Mg_0.15_Ni_3.8_	–	same	–	up	–	–	[155]
Polyaniline plating	La_0.8_Mg_0.2_Ni_2.7_Mn_0.1_Co_0.55_Al_0.1_	–	–	up	up	–	–	[156]
Magnetic annealing	La_0.67_Mg_0.33_Ni_3.0_	–	up	up	up	–	–	[157]
Chemical coprecipitation + metal reduction-diffusion	La_0.67_Mg_0.33_Ni_3.0_	–	–	–	–	–	Produces multi-phase structure	[158]

### 2.4. Ti-Ni-Based Alloys

The Ti-Ni-based system was the first MH alloy used in NiMH batteries in the early 1970 s. Although its development was interrupted by the fast growth of AB_5_ alloy, the Ti-Ni system remains a popular research topic due to its low cost, high hydrogen storage capacity, and fast activation. Two types of Ti-Ni binary alloys, TiNi and Ti_2_Ni, are able to absorb large amounts of hydrogen and are candidates as negative electrode material in NiMH batteries. TiNi alloy exhibits polymorphism, which is the basis of its outstanding shape memory property that is fully utilized in many industrial applications. Upon cooling, TiNi transforms from a B2 cubic structure (austenite) to a B19’ monoclinic structure (martensite). Early electrochemical studies on TiNi alloy demonstrated great activation performance and an electrochemical discharge capacity of 210 to 250 mAh·g^−1^ [159,160]. Compared to TiNi, Ti_2_Ni MH alloy had higher hydrogen storage capacity due to its higher content of hydride forming Ti in the formulation, but its electrochemical capacity was only 170 mAh·g^−1^ with its stronger metal-hydrogen bond strength, an indicator of dehydriding difficulty [160]. By combining TiNi and Ti_2_Ni phases, the electrochemical capacity increases up to 320 mAh·g^−1^ [160,161] with the assistance of synergetic effect between the two phases. During hydrogen desorption, TiNi, which has better desorption kinetics, first dehydrides and contributes to the overall electrochemical capacity; then, the hydrogen stored in Ti_2_Ni phase is transferred internally into the dehydrided portion of TiNi and discharged through TiNi. Without the assistance of TiNi phase, the hydrogen stored in Ti_2_Ni phase cannot be released due to the stronger meta-hydrogen bond strength. However, the cycle stability of such a biphasic system suffers due to the corrosion and oxidation of Ti_2_Ni [159,162].

Most research efforts on TiNi alloys focused on elemental modifications on both A- and B-sites. Different material manufacturing procedures, such as mechanical alloying, annealing, and different quench rates, were introduced to study the effect of structure on overall electrochemical properties. Emami *et al.* investigated the influence of Pd by partially replacing Ni [163,164]. While the unit cell of TiNi is enlarged by the larger-sized Pd, the electrochemical capacity is reduced since the Pd-substitution increases the stability of TiNi intermetallic alloy and decreases the stability of their hydrides according to the electronic calculations. Recently, a study on substituting Ni in TiNi with various modifiers revealed that Fe, Co, and Cr increase the electrochemical capacity up to 400 mAh·g^−1^ with good activation performance [165]. Zhao *et al.* fabricated amorphous Ti_2_Ni alloy by solid-state sintering and ball milling [166], and the resulting material shows improved cycle stability but also lower capacity compared to crystalline Ti_2_Ni. Annealing treatment was then proposed in addition to sintering and ball milling, which results in a thicker oxide layer on the surface of amorphous Ti_2_Ni and improves both capacity and charge retention of the alloy [167]. In order to increase the capacity, Zhao *et al.* developed another fabrication method for Ti_2_Ni: induction melting, ball milling, and annealing [162]. The product is amorphous and nanocrystalline in nature and demonstrates reasonable capacity and good cycle stability; however, the highest reported capacity of 363 mAh·g^−1^ for Ti_2_Ni MH alloy is obtained at higher temperatures while cycle stability suffers. Zr-substitution for Ti was used as another way to potentially increase capacity and improve cycle stability of amorphous Ti_2_Ni [168]. Zr was reported to destabilize amorphous Ti_2_Ni phase and promote TiNi phase when partially substituting Ti in ball-milled Ti_2_Ni from elemental powders [169]. An amorphous Ti_3_Ni_2_ alloy was prepared to combine advantages from TiNi and Ti_2_Ni, and although its capacity was lower than the crystalline form, cycle stability was much improved [170]. 

Ti-based icosahedral quasicrystal MH alloy, which has a crystallographically disallowed five-fold rotational symmetry, has only recently been studied for NiMH battery applications. The icosahedral phase is believed to contain much higher densities of tetrahedral interstitial sites compared to normal crystals [171]. Since hydrogen atoms enter favorably into tetrahedral sites, the icosahedral phase can absorb a large amount of hydrogen. Two recent studies, with and without milling after melt-spin, were performed varying the Zr/Ni ratio in Ti_45_Zr_38_Ni_17_[172,173]. All alloys consisted of 100% or close to 100% icosahedral phase and demonstrated that higher levels of Ni increase capacity up to 86 to 88 mAh·g^−1^; however, this value is dramatically lower than the theoretical capacity [172,173]. By changing the Zr/Ti ratio in Ti_45_Zr_30_Ni_25_, it was shown that higher Zr deteriorates capacity. Also with annealing, an alloy that has been arc-melted and mechanically alloyed has higher capacity (up to 130 mAh·g^−1^) compared to an unannealed amorphous alloy [174]. Hu *et al.* improved capacity up to 278 mAh·g^−1^ with decent cycle stability by adding Mn in a melt-spun TiVNi-based alloy [175]. The effect of Sc-addition was reported by the same group in TiVNi-based quasicrystal [176], with extended cycle life.

### 2.5. Mg-Ni-Based Alloys

Due to Mg’s abundance, low cost, light weight, and high hydrogen storage capacity (2200 mAh·g^−1^ theoretical electrochemical capacity [9]), Mg-based MH alloy continues to be an interesting research topic and a strong candidate for the negative electrode material of NiMH batteries. In order to be applicable for room temperature battery operation, a stoichiometry of Mg:Ni close to 1:1 is required. However, the MgNi intermetallic compound does not exist on the Mg-Ni binary phase diagram. Therefore, non-equilibrium fabrication methods, such as mechanical alloying, RF sputtering, laser ablation, and melt-spin, are often used to prepare MgNi alloys. Such material usually has a mixed structure of amorphous and nanocrystalline character. Despite the high capacity that MgNi alloy offers, its sluggish hydriding/dehydriding kinetics and poor corrosion resistance in alkaline media prevent MgNi from use in practical application. Various types of modifications to the MgNi system were studied to improve the overall electrochemical performance, such as replacements of A- (by rare earth, transition metals, or others) and B-sites (by transition metals), combinations of different fabrication procedures, and surface treatments [177]. Table 5 illustrates recent elemental substitution efforts on MgNi-based alloy. By varying the ball milling time, Anik *et al.* found that 15 h was sufficient to obtain the amorphous/nanocrystalline state [178,179]; however, 25 h was required to incorporate all Ni into the main MgNi phase. Electrochemical discharge capacity is also influenced by the milling period. The same report demonstrated that capacity increased sharply with up to 15 h milling time, stabilized with up to 25 h milling time, and decreased with further increase in milling time. Based on the role of each constituent element, a series of systematic elemental substitutions was conducted [178,179,180,181]. Anik *et al.* reported an improvement in electrochemical performance with Mg_0.80_Ti_0.15_Al_0.05_Zr_0.05_Ni_0.95_ alloy (420 mAh·g^−1^ and 90% capacity retaining rate at the 20th cycle) compared to the base MgNi alloy (495 mAh·g^−1^ and 35% capacity retaining rate at the 20th cycle) [178,181]. The pulverization mechanism of MgNi was investigated by in-situ monitoring of hydride-dehydride cycles using acoustic emission technique coupled with electrochemical measurement [182,183]. Unlike most cases where pulverization is caused by volume expansion and contraction with repeated hydrogenation, mechanically alloyed MgNi consists of porous agglomerates made up of many particles cold welded together, which are likely to be easily broken down by the mechanical action of hydrogen bubbles during hydrogen evolution. Hydrogen combustion synthesis was employed as another MgNi fabrication method; and with the help of subsequent ball milling and surface protection from NAFION coating, a 400% increase in capacity could be achieved [184].

**Table 5:** Summary of recent research on electrochemical property improvement for mechanically alloyed MgNi MH alloys.

Substitution	Alloy formula	Range of *x*	Capacity	Cycle life	Comment	Reference
Ti	Mg_1−*x*_Ti*_x_*Ni	0 to 0.2	up then down	up	As the Ti/Mg ratio increases, surface charge transfer resistance increases	[178,180]
Ti	Mg_0.7_Ti_0.3_Ni	–	down	up	–	[185]
Ti	Mg_1−*x*_Ti*_x_*Ni	0 to 0.1	down	up	Reduces pulverization	[186]
Zr	Mg_1−*x*_Zr*_x_*Ni	0 to 0.2	up then down	up	–	[178]
La	Mg_0.7_Ti_0.225_La_0.075_Ni	–	down	up	Further improves corrosion resistance	[185]
Al	Mg_1−*x*_Al*_x_*Ni	0 to 0.2	down	up	–	[178]
Al	Mg_0.9_Ti_0.1_NiAl*_x_*	0 to 0.05	down	up	Reduces pulverization	[187]
B	Mg_1−*x*_B*_x_*Ni	0 to 0.2	down	same	–	[178]
Pd	Mg_1−*x*_Pd*_x_*Ni	0 to 0.2	down	up	Surface charge transfer resistance decreases and then increases	[179]
Pd	Mg_0.9_Ti_0.1_NiAl_0.05_Pd*_x_*	0 to 0.1	down	up	Increases HRD	[187]
Pd	Mg_50_Ni_50−*x*_Pd*_x_*	0 to 5	down	up	–	[188]
Mg/Ni	Mg_0.85+*x*_Ti_0.15_Ni_1.0−*x*_	0 to 0.1	up	down	–	[180]

Mg_2_Ni is another Mg-Ni system that has been extensively studied for its potential applications in hydrogen storage and NiMH battery. With the higher content of hydride-former in the Mg_2_Ni formulation, it is capable of storing a greater amount of hydrogen compared to MgNi; however, Mg_2_Ni’s discharge kinetics and corrosion resistance are worse. Nanocrystalline/amorphous structure was shown to improve hydriding/dehydriding kinetics [189]; such structure can be achieved by mechanical alloying and melt-spin fabrication techniques or elemental substitution. Although the exact effect of melt-spin cooling rates depends on the type of elemental substitution, a higher cooling rate was frequently found to be beneficial in enhancing electrochemical properties and cycle stability [190,191,192,193,194,195,196,197]. Furthermore, the length of milling time plays an important role in varying the performances of mechanically alloyed materials [198,199]. Melt-spin in magnetic field has been reported to greatly improve both capacity and stability [200]. Zhu *et al.* provided a comparison among the influences of various processing methods on electrochemical characteristics [201]. Table 6 summarizes the recent elemental substitution efforts on the Mg_2_Ni system. It should be noted that besides the Mg-Ni MH systems, other Mg-based alloys, such as MgTi [202] and MgAl [203] systems doped with Ni, were also investigated for their electrochemical properties.

**Table 6:** Summary of recent research on electrochemical property improvement for Mg_2_Ni MH alloys. MS: melt-spin, MSM: melt-spin in magnetic field, HCS: hydriding combustion synthesis, BM: ball milling, MSP: magnetron sputtering.

Substitution/ Addition	Process	Alloy formula	Range of *x*	Capacity	HRD	Cycle life	Comment	Reference
Co	MS	Mg_2_Ni_1−*x*_Co*_x_*	0 to 0.4	up	up	up	Promotes amorphous phase	[190,191,194,195]
Mn	MS	Mg_2_Ni_1−*x*_Mn*_x_*	0 to 0.4	up	up then down	up	Promotes amorphous phase	[192,194,195]
Cu	MS	Mg_2_Ni_1−*x*_Cu*_x_*	0 to 0.4	up	up then down	up	–	[193,194,195]
La	MS	Mg_2−*x*_La*_x_*Ni	0 to 0.2	–	up	up	Promotes amorphous phase	[196,197]
–	MSM	Mg_2_Ni	–	up	–	up	–	[200]
Co	HCS+BM	Mg_2.1−*x*_Co*_x_*Ni	0 to 0.1	down	down	up	–	[204]
Cr	HCS+BM	Mg_2.1−*x*_Cr*_x_*Ni	0 to 0.1	down	down	up	–	[204]
Nb	HCS+BM	Mg_2.1−*x*_Nb*_x_*Ni	0 to 0.1	down	down	up	–	[204]
Ti	HCS+BM	Mg_2.1−*x*_Ti*_x_*Ni	0 to 0.1	down	down	up	–	[204]
V	HCS+BM	Mg_2.1−*x*_V*_x_*Ni	0 to 0.1	down	down	up	–	[204]
Al	HCS+BM	Mg_2−*x*_Al*_x_*Ni	0 to 0.7	up then down	–	–	–	[199]
Ti	BM	Mg_2−*x*_Ti*_x_*Ni	0 to 0.5	up	–	up	–	[198]
B	BM	Mg_1.5_Ti_0.3_Zr_0.1_Al_0.1_Ni	–	–	–	–	Compares to others in [205]	[205]
C	BM	Mg_1.5_Ti_0.3_Zr_0.1_Al_0.1_Ni	–	–	–	–	Compares to others in [205]	[205]
Fe	BM	Mg_1.5_Ti_0.3_Zr_0.1_Al_0.1_Ni	–	–	–	–	Compares to others in [205]	[205]
Pd	BM	Mg_1.5_Ti_0.3_Zr_0.1_Al_0.1_Ni	–	–	–	–	Compares to others in [205]	[205]
Al	BM	Mg_1.5_Ti_0.3_Zr_0.1_Al_0.1_Ni	–	–	–	–	Compares to others in [205]	[205]
Al	BM	Mg_2−*x*_Al*_x_*Ni	0 to 0.25	up	–	–	–	[206]
Multiwalled carbon nanotubes	BM	(MgAl)_2_Ni	–	up	–	–	–	[206]
Al	MSP	Mg_2−*x*_Al*_x_*Ni	0 to 0.3	up then down	–	–	Improves corrosion resistance	[207]

### 2.6. Laves Phase-Related BCC Solid Solutions

**Figure 5:**
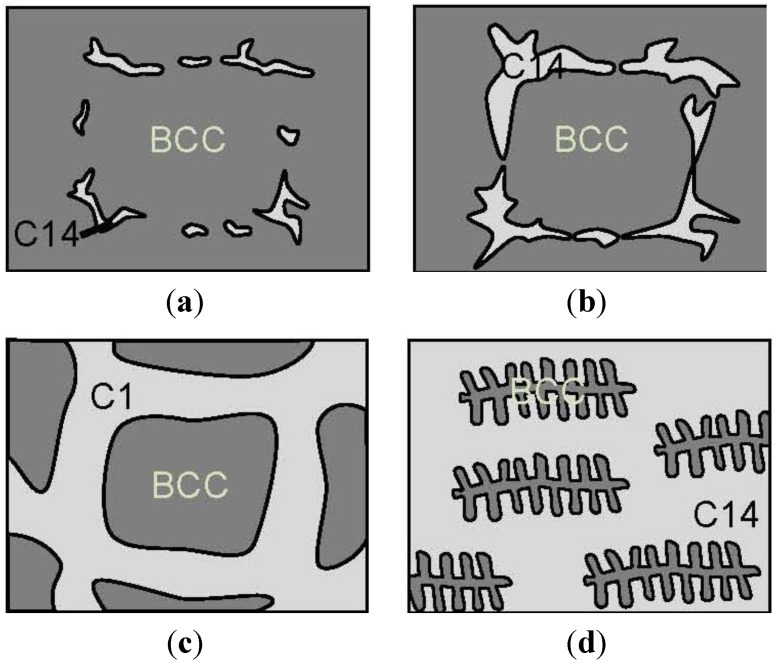
Schematics of microstructure evolution of a series of Laves phase-related body-centered-cubic (BCC) solid solution alloys as the C14 phase abundance increases (from Figure 5a–d).

“Laves phase-related body-centered-cubic (BCC) solid solution” [208] is an interesting MH alloy with a general formula of AB*_x_*, where A is from Group 4A (mainly Ti), B is from Group 5A, 6A, and 7A (mainly V), and x is between 1 and 6. It has a unique two-phase microstructure composed of a BCC phase and a Laves phase (mostly C14). Microstructure evolution as a function of C14/BCC ratio is constructed based on literature review and presented in Figure 5. During solidification, the high V BCC phase solidifies first and form a 3D framework and the rest of the liquid turns into C14 phase, which is also a complementary 3D framework, and such phenomena produces the following evolution in microstructure with the increase in C14/BCC ratio: C14 starts to appear at the grain boundary (Figure 5a) → sections of C14 phase start to connect and form a 3D network (Figure 5b) → BCC phase forms 3D framework and the cross-section is composed of isolated islands embedded in C14 matrix (Figure 5c) → BCC phase forms fish-bone type of inclusions (Figure 5d). The high density of phase boundaries contributes to the hydrogen storage properties in two ways: the promotion of a synergetic effect between BCC (high hydrogen storage capability) and C14 phases (better absorption kinetics and easy formation due to its brittleness), and the formation of a coherent and catalytic interface in between. 

**Figure 6:**
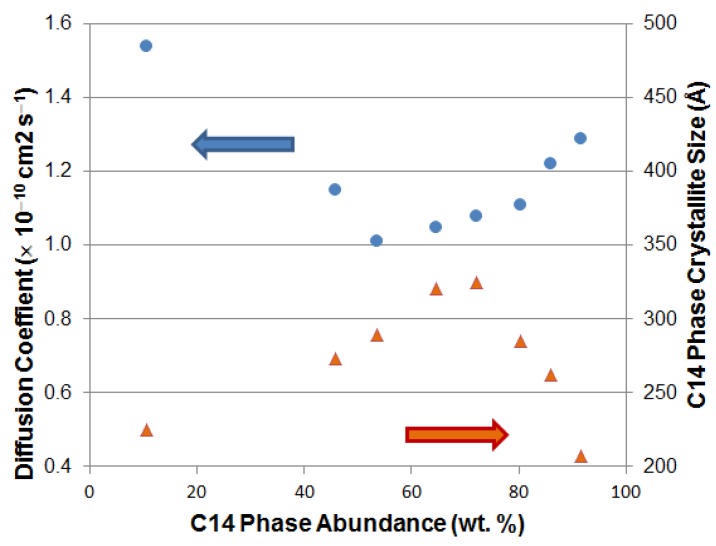
Plots of diffusion constant and crystallite size of the C14 phase determined by FWHM from XRD analysis as functions of C14 phase abundance for a series of Laves phase-related BCC solid solution alloys. The bulk transport is enhanced when the grain size is small and much of the boundary interface is available to contribute to the synergetic effect.

While Chai *et al.* claimed the best ratio of BCC:C14 is 1:1 for optimized results [209], Guéguen *et al.* showed that only a small amount of C14 is needed as a catalyst for the gaseous phase storage [210]. In order to study the correlation between the ratio and electrochemical properties of the alloy, we fabricated a series of Laves/BCC alloys with the Laves phase abundance from 10.5 to 91.6 wt %; their bulk diffusion coefficients and crystallite sizes are plotted in Figure 6. When one phase dominates, the crystallite size is smaller and the diffusion coefficient is larger. Therefore, a small but sufficient amount of secondary phase is preferable in the multi-phase MH alloy system. In addition, a BCC/C14 multi-phase alloy, reported by Wang and Ning, shows an electrochemical capacity of 450 mAh·g^−1^, but degradation through cycling is severe [211]. The BCC/C14 alloy was remelted with 10 wt % LaNi_3_ [212] and up to 10 wt % LaNi_5_ [213,214] by arc melting, and the activation behavior, HRD, low-temperature performance, and cycle life are all improved due to the synergetic effect between main and secondary phases. BCC/Ti-Ni-based multi-phase systems were also fabricated previously [117,215]. The existence of the TiNi secondary phase enhances HRD and cycle life and shows the highest capacity of 470 mAh·g^−1^ [215]. Recently, an anneal-and-quench method [216] has been implemented to prepare BCC/C14 alloys. Furthermore, quasicrystal-included alloys have also been investigated by Liu *et al.* [217,218,219,220,221], and capacity, HRD, and cycle stability at the 30th cycle up to 422 mAh·g^−1^, 88%, and 81%, respectively, were reported.

### 2.7. Zr-Ni-Based Alloys

Since the AB_2_ intermetallic compound does not exist on the Zr-Ni binary phase diagram, one or more secondary non-Laves Zr*_x_*Ni*_y_* phases composed of neighboring phases of AB_2_ are often observed when fabricating a Zr-Ni-based MH alloy with an AB_2_ composition. Between the working main phases and the catalytic secondary phases, a synergetic effect arises and contributes positively to the overall electrochemical performance. Although these minor phases do not have an appropriate metal-hydrogen bond strength when compared to the AB_2_ stoichiometry, it is essential to evaluate various Zr*_x_*Ni*_y_* alloys in order to gain better understanding of their roles in AB_2_ MH alloys and potentially develop alternative rare earth-free MH alloys for NiMH battery through composition modification. A systematic examination of Zr_8_Ni_21_, Zr_7_Ni_10_, Zr_9_Ni_11_, and ZrNi alloys correlating composition, structure, gaseous phase hydrogen storage, and electrochemical properties was recently reported by Nei *et al.* [77]. Annealing is detrimental to the electrochemical capacity of Zr*_x_*Ni*_y_* alloys due to the elimination of minor phases, similar to the annealing effect on AB_2_ alloy. Furthermore, the electrochemical capacity maximizes at Zr_7_Ni_10_, which demonstrates that both hydrogen desorption/discharge rate and theoretical maximum hydrogen storage determined by the Zr/Ni ratio influence the electrochemical discharge capacity. Among all, Zr_8_Ni_21_ shows the highest HRD and easiest activation. Zr_7_Ni_10_ and Zr_8_Ni_21_ were selected for further composition modification to improve their electrochemical properties [76,222,223,224,225,226], and the results are summarized in Table 7. One of the drawbacks of AB_2_ alloy compared to AB_5_ alloy is its lower HRD. For the purpose of increasing HRD in the Zr-based alloys, alloys with higher B/A ratios, such as ZrNi_5_ [26] and Zr_2_Ni_7_ [25], become more attractive. Performances of V-modified ZrNi_5_ and Zr_2_Ni_7_ are summarized in Table 7. Although their capacities are too low for practical application (≤177 mAh·g^−1^), their bulk hydrogen diffusion properties are superior to those of existing AB_5_, AB_2_, and A_2_B_7_ alloys. With further modification, electrochemical performances of ZrNi_5_ and Zr_2_Ni_7_ alloys are expected to improve.

**Table 7:** Summary of recent works on modification on Zr*_x_*Ni*_y_* MH alloys.

Substitution	Alloy formula	Range of *x*	Capacity	HRD	Comment	Reference
Ti	Ti*_x_*Zr_7−*x*_Ni_10_	0 to 2.5	–	up	Activation becomes easier Ti_1.5_Zr_5.5_Ni_10_ has good combination of capacity and HRD, 204 mAh·g^−1^ and 79%	[76]
V	Ti_1.5_Zr_5.5_V*_x_*Ni_10−*x*_	0 to 3.0	up	down	Main phase shifts from Zr_7_Ni_10_ to C14 Ti_1.5_Zr_5.5_V_0.5_Ni_9.5_ with Zr_7_Ni_10_-predominant structure has good combination of capacity and HRD, 242 mAh·g^−1^ and 80%	[222]
Cr	Ti_1.5_Zr_5.5_V_0.5_(Cr*_x_*Ni_10−*x*_)_9.5_	0.1 to 0.2	down	down	Main phase shifts from Zr_7_Ni_10_ to Zr_9_Ni_11_ to C14	[223,224]
Mn	Ti_1.5_Zr_5.5_V_0.5_(Mn*_x_*Ni_10−*x*_)_9.5_	0.1 to 0.2	up	up	Main phase shifts from Zr_7_Ni_10_ to Zr_9_Ni_11_ to C14	[223,224]
Fe	Ti_1.5_Zr_5.5_V_0.5_(Fe*_x_*Ni_10−*x*_)_9.5_	0.1 to 0.2	up	down	Main phase shifts from Zr_7_Ni_10_ to C15.	[223,224]
Co	Ti_1.5_Zr_5.5_V_0.5_(Co*_x_*Ni_10−*x*_)_9.5_	0.1 to 0.2	down	down	Main phase shifts from Zr_7_Ni_10_ to Zr_9_Ni_11_ to C15	[223,224]
Cu	Ti_1.5_Zr_5.5_V_0.5_(Cu*_x_*Ni_10−*x*_)_9.5_	0.1 to 0.2	down	down	Main phase stays Zr_7_Ni_10_	[223,224]
Al	Ti_1.5_Zr_5.5_V_0.5_(Al*_x_*Ni_10–*x*_)_9.5_	0.1 to 0.2	down	down	Main phase shifts from Zr_7_Ni_10_ to C14	[223,224]
Mg	Zr_8_Ni_19_Mg_2_	–	down	down	Main phase shifts from Zr_8_Ni_21_ to tetragonal Zr_7_Ni_10_	[225,226]
Al	Zr_8_Ni_19_Al_2_	–	up	down	Main phase shifts from Zr_8_Ni_21_ to tetragonal Zr_7_Ni_10_	[225,226]
Sc	Zr_8_Ni_19_Sc_2_	–	down	down	Main phase shifts from Zr_8_Ni_21_ to tetragonal Zr_7_Ni_10_	[225,226]
V	Zr_8_Ni_19_V_2_	–	down	down	Main phase shifts from Zr_8_Ni_21_ to Zr_2_Ni_7_	[225,226]
Mn	Zr_8_Ni_19_Mn_2_	–	down	down	Main phase shifts from Zr_8_Ni_21_ to Zr_2_Ni_7_	[225,226]
Co	Zr_8_Ni_19_Co_2_	–	down	down	Main phase shifts from Zr_8_Ni_21_ to Zr_2_Ni_7_	[225,226]
Sn	Zr_8_Ni_19_Sn_2_	–	down	up	Main phase shifts from Zr_8_Ni_21_ to Zr_2_Ni_7_ Annealed Zr_8_Ni_19_Sn_2_ is Zr_8_Ni_21_-structured	[225,226]
La	Zr_8_Ni_19_La_2_	–	up	down	Main phase shifts from Zr_8_Ni_21_ to orthorhombic Zr_7_Ni_10_	[225,226]
Hf	Zr_8_Ni_19_Hf_2_	–	up	down	Main phase shifts from Zr_8_Ni_21_ to orthorhombic Zr_7_Ni_10_	[225,226]
V	ZrV*_x_*Ni_4.5−*x*_	0 to 0.5	up	down	Main phase shifts from ZrNi_5_ to monoclinic Zr_2_Ni_7_	[26]
V	ZrV*_x_*Ni_3.5−*x*_	0 to 0.9	up then down	up	Main phase shifts from monoclinic Zr_2_Ni_7_ to cubic Zr_2_Ni_7_	[25]

### 2.8. Other Alloy Systems

It is nearly impossible to simultaneously meet all the requirements for a specific electrochemical application using one MH alloy. For example, an alloy with high capacity usually has lower HRD, and good activation behavior usually indicates shorter cycle life. Therefore, the development of composite MH alloys, which contain two or more hydrogen storage materials/intermetallic compounds/elements, can potentially combine the advantages of constituted alloys. Recent composite alloy studies include BCC related alloys modified by AB_2_ (Section 2.6), AB_5_ [227], LaNi_5_ [213,214], ZrV_2_ [220], A_2_B_7_-type [217], LaNi_3_ [212], or other BCC [218], A_2_B_7_-type alloy modified by AB_5_ [228], MgNi alloy modified by Ti(NiCo) [229], Mg_2_Ni alloys modified by TiNi, TiFe [230], (MgMn)_2_Ni [231], (MgAl)_2_Ni [232], Co, or Ti [233], and are summarized in Table 8.

Researchers have ventured out of well-understood MH alloy systems in order to develop a novel material that fulfills all requirements of electrochemical applications. *R*_6_*T*_23_ systems (*R* = Gd, Ho, *T* = Mn, Fe) were recently studied. He *et al.* performed B-site substitution using Co on Ho_6_Fe_23_ and found that the alloys have Th_6_Mn_23_-type structure [234]. As the level of Co increases, activation, capacity (443 mAh·g^−1^ at 150 mA·g^−1^), cycle stability, and HRD all improve. Electrochemical results were also reported on the Gd-Co-Mn system, and a promising capacity of 377 mAh·g^−1^ at 150 mA·g^−1^ was obtained [235]. The HRD of this type of alloy, however, is relatively low and in the range of 50% to 60%.

**Table 8:** Summary of recent research on composite alloys. BM: ball milling, AM: arc melting, ERM: electric resistance melting, IEC: isothermal evaporation casting.

Addition	Process	Base alloy	Addition level	Phase distribution	Capacity	HRD	Cycle life	Reference
MmNi _3.99_Al_0.29_Mn_0.3_Co_0.6_	BM	Ti_0.32_Cr_0.43–*x*–*y*_V_0.25_Fe*_x_*Mn*_y_*	0 to 20 wt %	BCC	up	–	–	[227]
LaNi_5_	AM	Ti_0.10_Zr_0.15_V_0.35_Cr_0.10_Ni_0.30_	0 to 10 wt %	BCC+C14+Zr-rich	up then down	up	up	[213,214]
ZrV_2_	BM	Ti_1.4_V_0.6_Ni	0 to 20 wt %	quasicrystal+Ti_2_Ni+BCC+C14+C15	up	up	up	[220]
La_0.65_Nd_0.12_Mg_0.23_Ni_2.9_Al_0.1_	BM	Ti_1.4_V_0.6_Ni	0 to 20 wt %	quasicrystal+Ti_2_Ni+BCC+LaNi_5_+PuNi_3_	same	up	down	[217]
LaNi_3_	AM	Ti_0.10_Zr_0.15_V_0.35_Cr_0.10_Ni_0.30_	0 to10 wt %	BCC+C14+Zr-rich	up	up	up	[212]
Ti_15_Zr_18_V_18_Ni_29_Cr_5_Co_7_Mn	BM	Ti_1.4_V_0.6_Ni	0 to 40 wt %	quasicrystal+Ti_2_Ni+BCC+C14	up	up	up	[218]
La_0.377_Ce_0.389_Pr_0.063_Pr_0.171_Ni_3.5_Co_0.6_Mn_0.4_Al_0.5_	BM	Mm_0.80_Mg_0.20_Ni_2.56_Co_0.50_Mn_0.14_Al_0.12_	0 to 30 wt %	LaNi_5_+La_2_Ni_7_	down	up then down	up	[228]
TiNi_0.56_Co_0.44_	BM	MgNi	0 to 50 wt %	Amorphous MgNi	down		up	[229]
TiNi	BM	Mg_2_Ni	0 to 100 mol %	TiNi+Mg_2_Ni	down			[230]
TiFe	BM	Mg_2_Ni	0 to 100 mol %	TiFe+Mg_2_Ni	up			[230]
Mg_3_MnNi_2_	ERM+IEC	Mg_2_Ni	0 to 100 mol %	Mg_2_Ni → Mg_2_Ni+Mg_3_MnNi_2_→ Mg_3_MnNi_2_	up		up	[231]
Mg_3_AlNi_2_	ERM+IEC	Mg_2_Ni	0 to 100 mol %	Mg_2_Ni → Mg_2_Ni+Mg_3_AlNi_2_ → Mg_3_AlNi_2_	up		up then down	[232]
Co	BM	Mg_3_MnNi_2_	0 to 200 mol %	amorphous Mg_3_MnNi_2_	up		up	[233]
Ti	BM	Mg_3_MnNi_2_	0 to 200 mol %	amorphous Mg_3_MnNi_2_	up		up	[233]

## 3. Conclusions 

Hydrogen storage alloys for electrochemical application have been extensively studied for many years. We have presented a review of recent research activities on metal hydride alloys for nickel metal hydride battery and also provided an overview of the use of metal hydrides in other electrochemical applications. AB_5_ and AB_2_ alloys are very well established systems. In order to potentially dominate the future electric vehicle and stationary applications, self-discharge, low-temperature performance, and cycle stability become more important to study, and the trend of recent research reflects the efforts on improving the aforementioned properties. Superlattice A_2_B_7_-type alloy, which possesses the advantages of both AB_5_ and AB_2_ and low self-discharge capability, is likely to be the next generation of metal hydride alloy used as the negative electrode material in nickel metal hydride batteries and has attracted much attention. Although the Ti-Ni alloy system is difficult to process and has poorer high-rate performance, its much lower raw material cost makes the system one that merits further studies for improvement. The Mg-Ni alloy system holds great promise in achieving very high capacity, and recent research efforts have concentrated on improving its kinetics and cycle capability for the purpose of practical implementation. Laves phase-related BCC solid solution has high capacity; enhancing its stability is currently the most essential topic. The incorporation of quasicrystals by various fabrication methods remains an interesting subject. Zr-Ni alloy systems were systematically investigated in the last few years. Although their performance might not be satisfactory for electrochemical applications at the present time, further elemental modifications or use as a composite modifier can assist in realizing their potential. 
